# Influence of Temperature on the Performance of Carbon- and ATO-supported Oxygen Evolution Reaction Catalysts in a Gas Diffusion Electrode Setup

**DOI:** 10.1021/acscatal.3c01193

**Published:** 2023-05-22

**Authors:** Aline Bornet, Rebecca Pittkowski, Tobias M. Nielsen, Etienne Berner, Annabelle Maletzko, Johanna Schröder, Jonathan Quinson, Julia Melke, Kirsten M. Ø. Jensen, Matthias Arenz

**Affiliations:** † Department of Chemistry, Biochemistry and Pharmaceutical Sciences, University of Bern, Freiestrasse 3, 3012 Bern, Switzerland; ‡ Department of Chemistry, University of Copenhagen, Universitetsparken 5, 2100 Copenhagen, Denmark; § Department for Applied Electrochemistry, Fraunhofer-Institute for Chemical Technology ICT, Joseph-von-Fraunhofer Strasse 7, 76327 Pfinztal, Germany; ∥ Biochemical and Chemical Engineering Department, Aarhus University, Åbogade 40, 8200 Aarhus, Denmark

**Keywords:** PEM water electrolysis, oxygen evolution reaction, Ir-based nanoparticles, supported OER catalysts, GDE setup

## Abstract

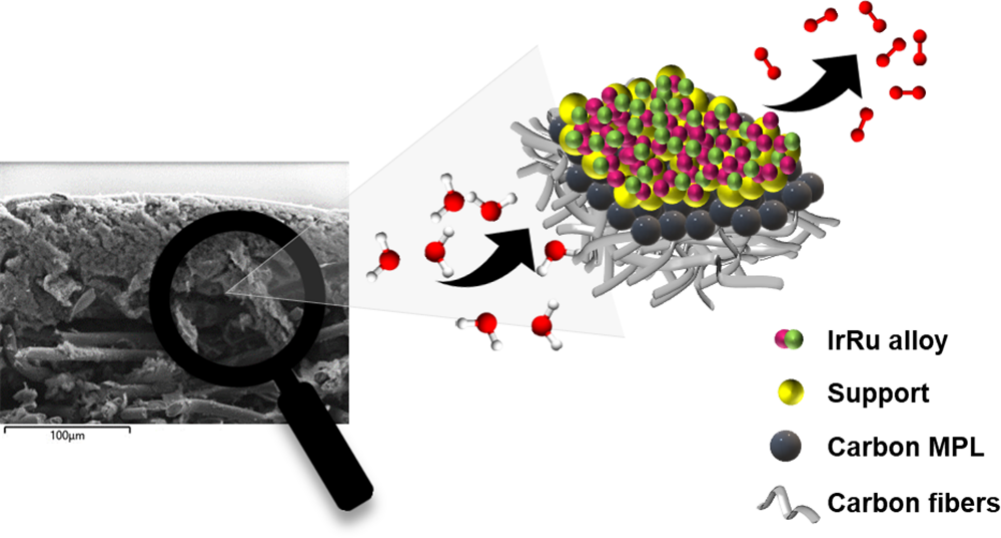

State-of-the-art industrial electrocatalysts for the oxygen evolution reaction (OER) under acidic conditions are Ir-based. Considering the scarce supply of Ir, it is imperative to use the precious metal as efficiently as possible. In this work, we immobilized ultrasmall Ir and Ir_0.4_Ru_0.6_ nanoparticles on two different supports to maximize their dispersion. One high-surface-area carbon support serves as a reference but has limited technological relevance due to its lack of stability. The other support, antimony-doped tin oxide (ATO), has been proposed in the literature as a possible better support for OER catalysts. Temperature-dependent measurements performed in a recently developed gas diffusion electrode (GDE) setup reveal that surprisingly the catalysts immobilized on commercial ATO performed worse than their carbon-immobilized counterparts. The measurements suggest that the ATO support deteriorates particularly fast at elevated temperatures.

## 1 Introduction

Hydrogen is broadly used in the chemical industry, and as of today, most of it is derived from natural gas. The year 2022 has shown that the large demand for natural gas leads to critical economical dependencies. As an alternative, the production of hydrogen from electrochemical water splitting using renewable energy may be a valuable strategy for a more sustainable future.^1−3^ This so-called green hydrogen can be used as a storage solution for surplus energy from renewable sources and thus can help to tackle the challenge of climate change.

Acidic proton exchange membrane water electrolyzers (PEMWEs) constitute an industrially relevant and viable technology for producing green hydrogen. Indeed, they have a compact design, can reach high current densities, and can generate high-pressurized hydrogen of high purity. In the case of energy storage, this green hydrogen can be later used in fuel cells.^1,2,4^

Catalyst development for the oxygen evolution reaction (OER) is one of the key aspects and bottlenecks to permit the PEMWE technology to be implemented at a large scale.^4,5^ Despite the high price and the scarcity of Ir and Ru,^4^ and although intensive efforts have been made to alleviate the need for these critical raw materials (CRMs),^6−11^ Ir- and IrRu-based catalysts remain the state-of-the-art materials for the acidic OER.^12^ Several approaches have been considered in order to reduce the use of CRMs in catalysts for the acidic OER. However, it is important to note that to be commercially viable, this CRM reduction needs to be calculated with respect to the converted power (hydrogen) and not only with respect to the catalyst composition. Therefore, an important strategy to maximize the dispersion (surface-to-mass ratio) of the CRMs is to tune the particle size and morphology of the Ir and/or Ru on the nanoscale. In the literature, many examples of this strategy can be found, *e.g.*, designing tailored shapes such as nanoparticles (NPs),^13−15^ nanowires,^16,17^ and nanodendrides^18^ or hollow structures like nanoframes,^19^ and nanoporous networks.^20^ Another strategy to increase their mass-related activity is to introduce other non-noble elements, typically transition metals such as Co, Ni, and Cu.^21−24^ Such multimetallic materials can be found in the form of core–shell structures,^24^ alloys,^21,22^ or composite materials.^25^ Furthermore, the introduction of support materials—state-of-the-art OER catalysts are unsupported—may be a viable strategy to enhance the mass-related catalytic performance.^5^ The latter strategy presents the advantage of a reduced catalyst loading thanks to a better NPs dispersion. Hence, the utilization of the catalyst is improved by increasing the amount of exposed active sites, and therefore by increasing the electrochemically active surface area (ECSA) of the catalysts.^5,26,27^ In fuel cell applications, carbon black is commonly used as a support material due to its low cost, high surface area, and good conductivity. However, it is well known that carbon-based (C-based) supports are unstable under harsh OER conditions in acidic media.^20,27−29^ In fact, even at oxygen reduction reaction (ORR) conditions, C-based supports are only kinetically stable.^30−33^ Therefore, more recently, extensive studies have been conducted to maximize the surface area, stability, and conductivity of other support materials for OER catalysts. Among others, mesoporous conductive oxides such as antimony-doped tin oxide (ATO),^15,34^ tin-doped indium oxide (ITO),^35^ and fluorine-doped tin oxide (FTO)^35^ were considered promising support candidates.

To address the challenge of designing a cost-effective, highly dispersed catalyst, we herein present ultrasmall Ir and Ir*_x_*Ru*_y_* NPs deposited on a standard fuel cell carbon support (Kejten Black) as well as on commercially available ATO. The catalyst preparation was performed in two steps: first, the synthesis of surfactant-free, colloidal NPs in a low-boiling-point solvent^13,36^ and second, the immobilization on the support. This flexible approach allows a versatile catalyst design by varying several parameters independent of each other, in the present case the support material independent of NP composition. That is, the same NPs are studied on different support materials.^37^ The focus has been made on supported Ir and Ir_0.4_Ru_0.6_ NPs as, according to the density functional theory (DFT) calculations of Svane et al.,^38^ the latter corresponds to the optimum composition for the OER. The electrocatalytic activity of the prepared OER catalysts was studied using an in-house developed gas diffusion electrode (GDE) setup. This cell has been previously used for the oxygen reduction reaction (ORR)^39−41^ and recently optimized for OER studies.^42^ In this screening device, more realistic and practical conditions can be reached as compared with the conventional rotating disk electrode (RDE) setup. In particular, realistic catalyst loadings are studied (up to 1 mg cm^–2^), membranes can be introduced, and the operating temperature can be easily varied.^42,43^ In the following study, the influence of the support on the overall activity was probed at a high temperature (60 °C) and its applicability in real PEMWE was discussed.

## 2 Experimental Part

Chemicals and materials are listed in the SI.

### 2.1 Synthesis and Deposition on a Support

Ir and Ir_0.4_Ru_0.6_ catalysts were synthesized using a slightly modified protocol from Bizzotto et al.^13,36^ Ir NPs were obtained by mixing 2 mL of 20 mM IrCl_3_ solution in ethanol (EtOH) with 7 mL of 57 mM NaOH/EtOH solution. The resulting molar ratio of NaOH and Ir is 10. This solution mixture was placed in an oil bath at 85 °C for 10 min under reflux conditions and constant stirring at 300 revolutions per minute (rpm). The color change from yellow to green and then to light brown indicates the formation of colloidal NPs. Once the reaction was completed, the solution was left to cool down under constant stirring, leading to a stable colloidal dispersion. A corresponding procedure was used to synthesize Ir_0.4_Ru_0.6_ NPs. 1 mL of 20 mM IrCl_3_ in EtOH and 1 mL of 20 mM RuCl_3_ in EtOH were employed, and the reaction temperature increased to 95 °C, while the reaction time stayed the same. The color transition revealing the NP formation was brown to yellowish to dark brown. The synthesis of the three other compositions (nominal composition: Ir_0.66_Ru_0.33_, Ir_0.33_Ru_0.66_, Ru) can be found in the SI.

To immobilize the NPs on the support, either carbon Ketjen Black (C) or a commercially available SbO_2_-doped SnO_2_ (ATO) was dispersed in EtOH (1:2, mass (support):volume (EtOH)) using a horn sonicator (4 min, pulse: 1 s on/1 s off, amplitude: 30%). The freshly prepared NPs were then poured into the beforehand-dispersed support, and the mixture was further sonicated under the same conditions for 10 more minutes.

The solvent was removed by means of a rotary evaporator (120 rpm, room temperature (RT), 5 °C cooling system) under constant sonication. The catalyst was left overnight under the hood. A second step of rotary evaporator (25 rpm, water bath at 85 °C, 5 °C cooling system, 30 mbar, 4 h) was preferably performed to completely dry the catalyst and to remove any undesired, volatile side products.

### 2.2 Ink Preparation

A similar procedure to the one reported by Schröder et al.^42^ was used to prepare the ink. The as-synthesized catalyst was dispersed in a 3:1 volume ratio of Milli-Q water and isopropanol (IPA). 70 μL of 1 M KOH was added per 60 mL of ink. KOH was added to increase the homogeneity and to improve the stability of the ink.^44^ The ink concentration was 654 μg_metal_ mL^–1^. After 5 min of bath sonication at RT, 10 wt % of Nafion with respect to the catalyst (NPs and support) was added to the ink. Finally, the ink was sonicated for 5 more minutes at RT.

### 2.3 Electrode Preparation

Following the description of Yarlagadda et al.,^45^ a coated carbon gas diffusion layer (GDL) was placed between a sand core filter and a glass funnel (⌀ 3.7 cm) in a vacuum setup. 4.8 mL of the 654 μg_metal_ mL^–1^ ink was diluted with 12.11 mL of Milli-Q water and 45.92 mL of IPA to reach a water/IPA volume ratio of 1:3 (metal concentration of 0.05 mg_metal_ mL^–1^). After filtration, the catalyst layer was dried overnight in air. The obtained nominal loading was 0.292 mg_metal_ cm^–2^ (see Figure S1 and Table S1 for the loading determination of a 1 mg_metal_ cm^–2^ Ir/C sample).

Following the procedure reported by Schröder et al.,^42^ a 3 mm diameter disk was punched out of the 3.7 cm catalyst film. A centered hole (⌀ 3 mm) was punched out of a coated GDL (⌀ 2 cm), where the 3 mm catalyst disk was then placed. An activated Nafion membrane was placed on top of it (see Figure S2a), and the whole system was placed between a paper sheet and an aluminum foil. It was pressed between two Teflon blocks by applying 2.5 tons of force for 10 min. To create an unbroken conductive surface, a ⌀ 2 cm noncoated GDL was placed below the Nafion-functionalized GDL-pressed system.

### 2.4 Electrochemical Measurements

An electrochemical cell (see Figure S2), dubbed GDE setup, in a three-electrode configuration was used to test the performance of the catalyst. The freshly pressed 3 mm functionalized GDL was employed as the working electrode and a platinum mesh as the counter electrode. All potentials were measured with respect to a reversible hydrogen electrode (RHE). The measurements were performed using a potentiostat controlled with the software EC4DAQ version 2.44. Humidified (Milli-Q water) O_2_ was continuously flowing through the setup during the measurements. A flow rate between 50 and 60 mL min^–1^ was used for each measurement. 4 M HClO_4_ was used as the electrolyte in the upper polyether ether ketone (PEEK) compartment of the setup. The electrolyte was preheated at most 7 °C above the desired temperature (30, 40, or 60 °C). The aluminum-made faradaic cage was preheated to the desired temperature using a thermocouple-controlled heating plate. Before each measurement, two cyclic voltammograms (CVs) were recorded between 1.2 and 1.6 V at 10 mV s^–1^ to ensure the correct connectivity of the cell.

Catalyst activation was performed by holding the potential at 1.6 V for 5 min.

Activity experiments were conducted using the following two current density sequences:

- For Ir catalysts: 0.85, 0.85, 2.14, 4.28, 8.56, 17.12, 25.68, 38.53, 51.37, 68.49, 85.62, 128.43, 171.23, 299.66, 428.09, 856.17, 1712.35 mA mg_Ir_^–1^
- For Ir_0.4_Ru_0.6_ catalysts: 0.43, 0.43, 1.07, 2.14, 4.28, 8.56, 12.84, 19.26, 25.68, 34.25, 42.81, 64.21, 85.62, 149.83, 214.04, 428.08, 856.16 mA mg_IrRu_^–1^

The solution resistance was determined online using an AC signal of 5 kHz with an amplitude of 1–10 mA.

For each temperature, triplicate samples were measured using a fresh electrode and new electrolyte: the first measurement followed the complete sequence, while the two others were stopped at 299.66 mA mg_Ir_^–1^ and at 214.04 mA mg_IrRu_^–1^ for Ir and Ir_0.4_Ru_0.6_ catalysts, respectively.

Electrochemical results have been exported and analyzed with the software EC4View. The last 100 s of each i*R*-corrected current step were averaged for activity determination.

### 2.5 Conductivity Measurements

A test rig was built to measure the electrical conductivity σ of the support powders by compressing them between two gold-plated copper stamps, with an area *A* of 38.5 mm, at different pressures with a maximum of 11.29 MPa. A multimeter was used to apply a direct current and simultaneously measure the resistance *R*_Ω_. The thickness *t* of the sample was measured by a laser distance sensor and was used for the calculation of the electrical conductivity of the pellet by the following equation
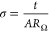
1

The measurements were repeated three times for the ATO support and two times for the C support.

### 2.6 Transmission Electron Microscopy (TEM)

TEM micrographs of the unsupported NPs were acquired with a Jeol 2100 operated at 200 kV. TEM micrographs of the supported NPs were acquired with Tecnai Spirit operated at 80 kV. The samples were prepared by drop-casting 10 μL of the ink on a grid and dried under air at RT. The mean size particle (diameter) of 150 particles was determined using the software ImageJ.

### 2.7 Small-Angle X-ray Scattering (SAXS)

*Ex situ* SAXS measurements were performed at the Paul Scherrer Institute (PSI), Switzerland, on the X12SA beamline, cSAXS, to assess the size change of the supported NPs before and after activation. The data were collected in a *q*-range of 0.0049–0.7198 Å^–1^ with a beam energy of 11.2 keV. The measurements were performed on pristine and activated 3 mm catalyst-functionalized GDL. The backgrounds corresponded to the supports without any NPs deposited on a GDL. Those were pristine and activated as well (the activation step is the same as the actual samples, see Section 2.4). All samples and backgrounds were measured with a Nafion membrane and were protected in Kapton tape.

The data analysis was performed using the software XSACT 2.4. The data were analyzed in the NPs module between 0.04 and 0.35 Å^–1^ for Ir_0.4_Ru_0.6_/C and Ir/ATO (both pristine and activated), between 0.045 and 0.28 Å^–1^ for Ir/C (pristine and activated) and activated Ir_0.4_Ru_0.6_/ATO, and between 0.045 and 0.31 Å^–1^ for pristine and Ir_0.4_Ru_0.6_/ATO. The model and the parameters for the calculation were the same for all samples, namely a spherical particle shape and a size distribution between 0.01 and 10 nm, with steps of 0.1 nm.

### 2.8 Energy-Dispersive X-ray Spectroscopy (EDX)

EDX measurements were performed on a Zeiss GeminiSEM 450 equipped with an EDX Photodetector Ultim Max 65 from Oxford Instruments to study the elemental composition of Ir_x_Ru_y_ catalysts and the possible Sb leaching of ATO-immobilized catalysts. The data were analyzed with the AZTec 4.2 software. To obtain only the atomic (atom %) ratio between Ir and Ru, other elements present in the sample were deconvoluted. The ratio between Ir and Ru was first determined from the ink. For that, about 3 × 10 μL was drop-cast onto a graphite foil. The samples were mounted on metal stubs with conductive, adhesive Cu tape. An accelerating voltage of 10 keV, a working distance of 8.5 mm, and probe currents between 400 and 500 pA were used as measuring parameters.

The ratio between Ir and Ru was determined a second time after the catalysts have been deposited on the GDL *via* vacuum filtration (see Section 2.3). The functionalized GDL was measured in a top-view mode. Cross-section mapping was also monitored onto functionalized GDL to identify the different layers and ensure the homogeneity of the catalyst layer.

Furthermore, the activated samples were measured in a top-view mode to identify any Ru leaching after the activation step (see Section 2.4). To activate the sample, the Nafion membrane was not pressed onto the functionalized GDL but simply deposited to allow its easier removal and avoid any catalyst layer destruction.

Finally, ATO-immobilized catalysts were analyzed postmortem to determine a possible Sb leaching. The samples were measured in a top-view mode.

### 2.9 Thermogravimetric Analysis (TGA)

A thermogravimetric analysis (TGA) instrument (Q500 V20.13, TA Instruments) was used to determine the metal loading on the 3 mm diameter GDE sample. An Ir/C sample of 1 mg_Ir_ cm^–2^ was used as a representative measurement. The sample was heated in an O_2_ atmosphere (O_2_ 5% in N_2_) from 25 °C (RT) to 1000 °C with a temperature ramp of 10 °C min^–1^. In the end, an isothermal step was held for 5 min. The sample was measured in the Danish Technological Institute (DTI), Denmark, *via* a send in service.

### 2.10 X-ray Absorption Spectroscopy (XAS)

*Ex situ* X-ray absorption near-edge structure (XANES) and extended X-ray absorption fine structure (EXAFS) measurements were carried out for the C-immobilized samples at the SuperXAS beamline of the Swiss Light Source (SLS) at PSI, Switzerland (storage ring current of 400 mA), *via* a send in service. The incident beam was collimated by a mirror (Rh-coated for Ir L_III_) and monochromatized with a liquid nitrogen-cooled channel-cut Si(111) monochromator. The measurements of the ATO-supported samples were performed at the ROCK beamline of the SOLEIL light source (storage ring current of 500 mA), France. The incident beam was collimated using a mirror with a 50 nm Ir coating and monochromatized with a Si(111) monochromator.

Energy calibrations were performed with simultaneously probed metal foils to reference the energies of the Ir L_III_-edge and the Ru K-edge positions. X-ray absorption spectra at the Ir L_III_-edge were collected in transmission mode, while Ru K-edge X-ray absorption spectra were measured in fluorescence mode. All spectra were collected in quick EXAFS mode (QEXAFS).^46−48^ The data were processed using ProQEXAFS for calibration, interpolation, normalization, and averaging (300 s of measurement on each sample). The leached samples were protected in Kapton tape.

The averaged XAS spectra were analyzed by using the Demeter software package. The raw spectra were energy aligned to a metal reference foil, background corrected, and normalized by the edge step. After conversion of the energy units (eV) into photoelectron wave number *k* units (Å^–1^), the resulting χ(*k*) functions of the XAS spectra were weighted with *k*^2^ and Fourier transformed to obtain pseudo-radial structure functions. The fits to the EXAFS spectra were performed in Artemis of the Demeter software package based on IFFEFIT.^49^ XAS spectra of the pure metal foils were used as references to estimate the amplitude reduction factors (S0^2^). The Ir L_III_-edge data were fitted in *R*-space, with a fitting weight of *k*^2^. The *k*-range for the Fourier transform was from 3 to 14 Å^–1^ with a fit window in an *R*-range of 1.1–3.0 Å. The Ru K-edge data were fitted in *R*-space, with a fitting weight of *k*^2^. The *k*-range for the Fourier transform was from 3 to 12 Å^–1^ with a fit window in an *R*-range of 1.0–3.0 Å.

### 2.11 Pair Distribution Function (PDF) Analysis

*Ex situ* synchrotron X-ray total scattering measurements were performed at the 11-ID-B beamline at the Advanced Photon Source (APS) and at the DanMAX beamline at MAXIV, with hard X-rays of 58.7 and 35.0 keV, respectively. At the APS beamline, the measurements were performed on pristine and activated 3 mm catalyst-functionalized GDLs. To isolate the scatting signal from the Ir and IrRu phases, data were collected for background subtraction. The backgrounds corresponded to the supports without any NPs deposited on a GDL. Those were pristine and activated (activation step similar to the actual samples, see Section 2.4). Scattering data from all samples and backgrounds were measured with a Nafion membrane and were protected in Kapton tape. At the DanMAX beamline, the measurements were performed in polyimide tubes filled with pristine catalyst powder or backgrounds. All diffraction patterns were collected in a wide-angle transmission geometry with 2D area detectors placed close to the sample. Fit2D,^50^ pyFAI,^51^ and Dioptas^52^ were used to calibrate experimental parameters from a calibrant material (CeO_2_ at APS, LaB_6_ at DanMAX) and to azimuthally integrate the diffraction images to 1D diffraction patterns. PDFgetX3^53^ and xPDFsuite^54^ were used to obtain the total scattering structure function, F(Q), which was sine Fourier transformed to obtain the PDF.

Modeling of the PDFs was carried out using PDFgui.^55^ The models used were face-centered cubic Ir and NaCl (*Fm3̅m*), and tetragonal IrO_2_ and ATO (*P4*_2_*/mnm*). Multiphase real-space Rietveld refinements were carried out, where scale factors, lattice constants (a, b, c), isotropic gaussian atomic displacement parameters, and spherical particle size parameters were refined. Measurement-specific resolution parameters, Q_broad_ and Q_damp_, were obtained by the refinement of the PDF data of a calibrant material, measured in the same geometry as the samples.

## 3 Results and Discussion

**Figure 1:**
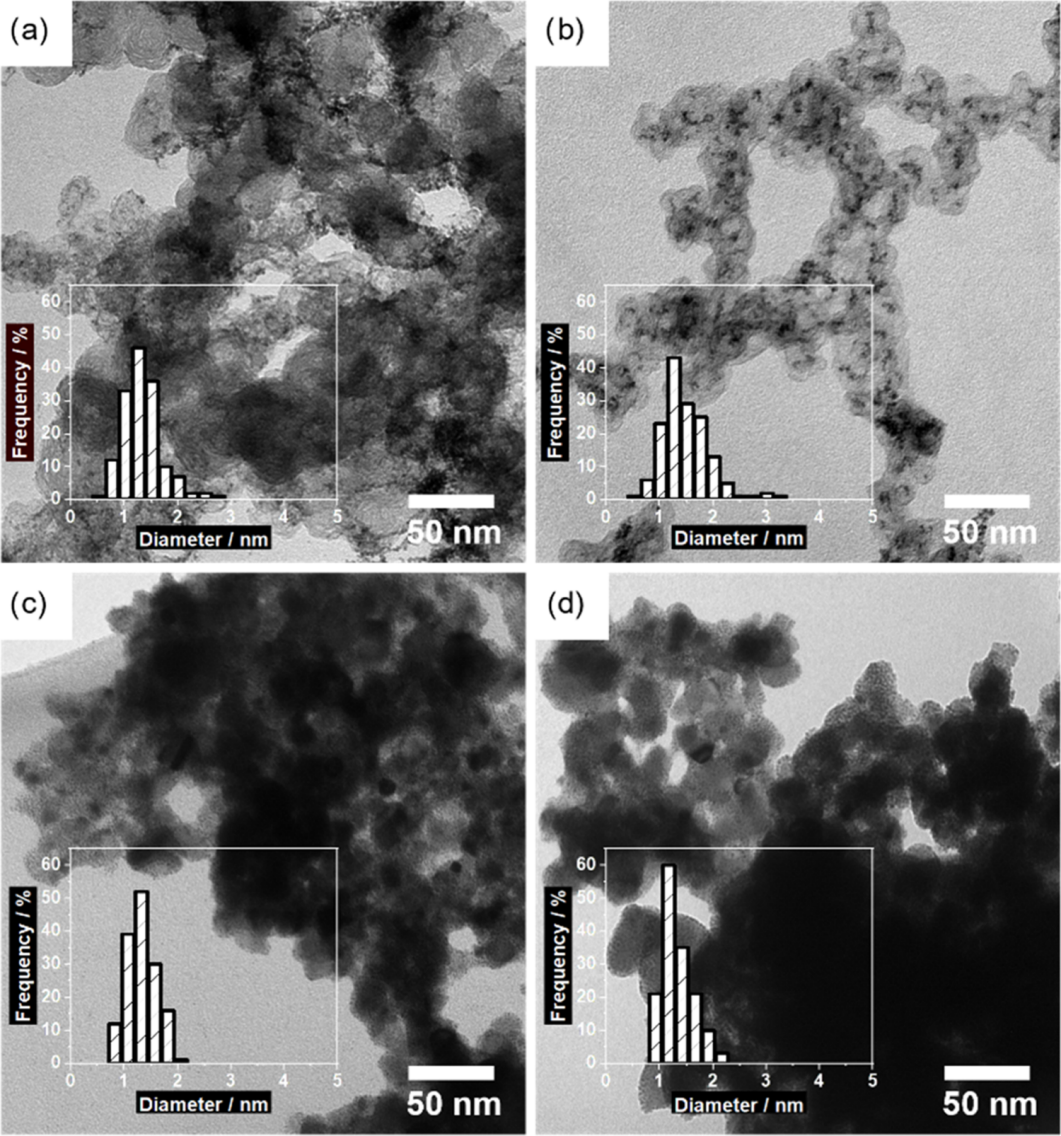
TEM micrographs and respective particle size distribution (insets) of Ir/C (a), Ir_0.4_Ru_0.6_/C (b), Ir/ATO (c), and Ir_0.4_Ru_0.6_/ATO (d).

Ultrasmall, *i.e.*, *ca*. 1.5 nm in diameter, pristine Ir and Ir*_x_*Ru*_y_* NPs were synthesized *via* a surfactant-free, colloidal route using only EtOH as a low-boiling-point solvent.^13,56,57^ The NPs were immobilized in a second step on two different commercially available supports, carbon Ketjen Black and ATO, at a nominal metal loading of 50 wt %. The straightforward synthesis approach allows for synthesizing a wide range of different compositions while keeping the particle size constant (see Figure S3). It also allows immobilizing the “same” NPs onto different supports, *i.e.*, NPs from the same batch. In the used two-step synthesis strategy, the support material is expected to have a negligible influence on the properties of the immobilized NPs,^37^ as the immobilization procedure does not involve any heating. This is supported by XAS data of Ir/C and Ir/ATO, in which both catalysts show overlapping spectra (Figure S4 and Table S2). While the colloidal NPs are metallic when synthesized, they slowly oxidize when exposed to air during immobilization and subsequent storage, as evidenced by the XAS spectra (Figure S5). The degree of oxidation, therefore, depends on the duration of storage (see Figure S5). It should also be noted that upon electrochemistry measurements, the NPs can be reduced again as shown in previous work.^13^ Nevertheless, the pristine samples are highly ordered and have a metallic core. PDF shows a pure fcc phase for Ir NPs, whereas Ir_0.4_Ru_0.6_ NPs can be fitted with either a pure fcc phase or a mixed fcc (as expected for Ir) and hcp phases (as expected for Ru), with similar accuracy (*R*_w_(fcc) = 0.65, *R*_w_(fcc + hcp) = 0.62) (Figure S6 and Table S3). However, EXAFS indicates alloying in the Ir_0.4_Ru_0.6_ NPs, *i.e.*, coordination between Ir and Ru atoms is seen (see Table S4). The morphology of the supported NPs was analyzed with a TEM, confirming spherical shape with a mean size of *ca*. 1.5 nm regardless of the composition or the support (see Figure 1 and Table S5). The micrographs reveal a slightly better dispersion of the NPs on the ATO support as compared with the C support, where small aggregates are formed.

**Figure 2:**
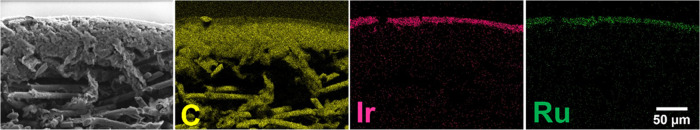
SEM/EDX cross-section mapping of C (yellow), Ir (pink), and Ru (green). The sample corresponds to a GDL functionalized with Ir_0.4_Ru_0.6_/C before activation.

As the active phase for the OER are oxides, the catalysts need to be activated before determining their catalytic activity.^13^ Therefore, the catalyst samples were electrochemically oxidized prior to each measurement. The activation leads to a particle growth of roughly twice the initial diameter as monitored by SAXS (see Figure S7 and Table S5). Assuming fully reduced pristine NPs, the determined growth in particle size due to oxidation is slightly less than expected (see discussion in the SI). This indicates that the NPs were not completely oxidized after the activation procedure. The same phenomenon was already observed by Minguzzi et al.,^58^ who highlighted the presence of both metallic and oxidic phases in Ir samples cycled up to 1.5 V *vs* RHE. To reinforce this hypothesis, PDF analysis of the total scattering of C-immobilized samples was carried out (see Figure S6) (the analysis of the ATO-immobilized was difficult due to the presence of the oxide support, see Figure S8), showing a small contribution from metallic phases, even after activation. The same is the case for the EXAFS data, which indicate metal–metal coordination of the activated samples as well (see Table S6). For the electrocatalytic measurements in the GDE setup, the catalyst was transferred onto a GDL by vacuum filtration. Figure 2 depicts a cross-sectioned SEM/EDX mapping of a representative catalyst film on a GDL. Starting from the bottom part to the top, the porous carbon fibers of the GDL, the microporous carbon layer (MPL), and the catalyst layer can be identified. The latter forms a homogeneous layer of about 12 μm thickness. No penetration of the catalyst sample into the GDL is observed, confirming a localized catalyst layer.

**Figure 3:**
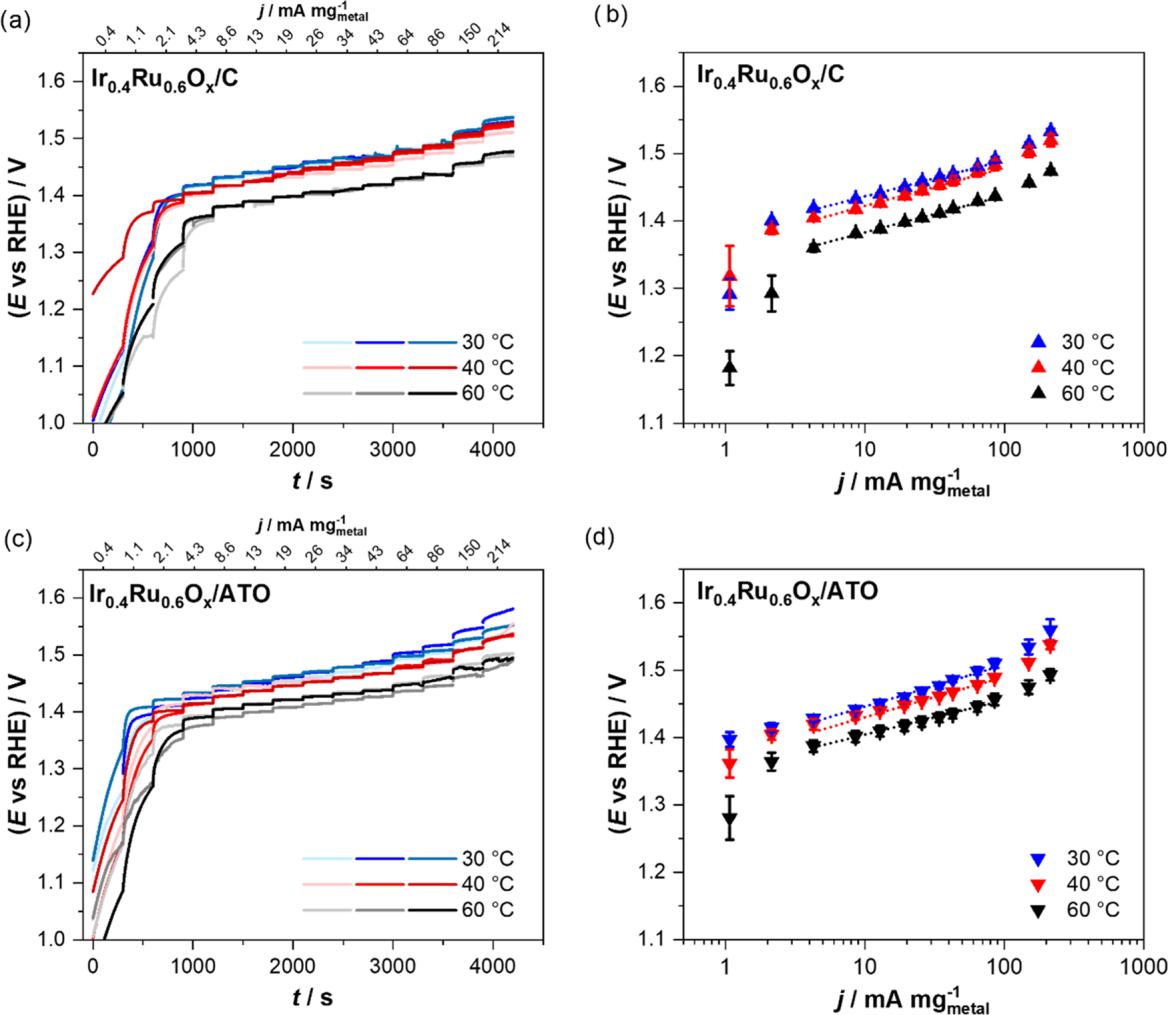
Electrocatalytic OER i*R*-corrected potential transients (a, c) and corresponding Tafel plots (b, d) of Ir_0.4_Ru_0.6_O*_x_*/C (upper row) and Ir_0.4_Ru_0.6_O*_x_*/ATO (lower row) at 30 (blue), 40 (red), and 60 °C (black). The error bars show the standard deviation of the three independent measurements. All measurements were performed in the GDE setup in an O_2_ atmosphere using 4 M HClO_4_ as an electrolyte. Nominal catalyst loading: 654 μg_metal_ cm^–2^.

The OER activity of the different catalysts was determined in galvanostatic measurements at steady-state conditions. Each sample was measured at least as triplicates using a fresh sample, and independent measurements were conducted at three different temperatures, *i.e.*, 30, 40, and 60 °C. Figure 3a,c depicts the i*R*-corrected raw data with the different galvanostatic steps and the achieved reproducibility among the individual measurement for Ir_0.4_Ru_0.6_O*_x_*/C and Ir_0.4_Ru_0.6_O*_x_*/ATO catalysts. The measurements for the pure Ir catalysts are reported in the SI (Figure S9) and demonstrate equally good reproducibility. It is seen that at the first two galvanostatic steps, no steady-state behavior was reached. Instead, the recorded potential increased with time in this initial galvanostatic step. This observation is in agreement with incomplete oxidation after the activation procedure. Prior to the OER measurements, the samples were activated by holding the potential at 1.6 V for 5 min. Despite this relatively high activation potential, the electrocatalytic data reach steady-state behavior only at the subsequent current steps. Then, a linear Tafel behavior is observed between *ca.* 4 and 85 mA mg_metal_^–1^ in all four cases (see Figures 3b,d and S10 and Table S7). It should be noted that taking the measurements before complete activation into account in the activity evaluation would lead to an overestimation of the OER activity as the recorded current is due to a mixture of OER and metal oxidation current. While such a behavior can be easily identified in steady-state measurements (potentiostatic or galvanostatic) applied here, it is very difficult to discern in the more commonly applied potentiodynamic cyclic or linear sweep voltammetry.

In our measurements, each catalyst demonstrates similar i*R*-corrected Tafel slopes at three different temperatures (see Table S7). At 30 °C, the Tafel slopes are in the range of 53–62 mV dec^–1^, which lies within the data reported in the literature for Ir-based catalysts.^15,18,59,60^ At higher current densities (>100 mA mg_metal_^–1^), deviations from the linear behavior are seen, which presumably are related to the formation of oxygen bubbles.^61^

**Figure 4:**
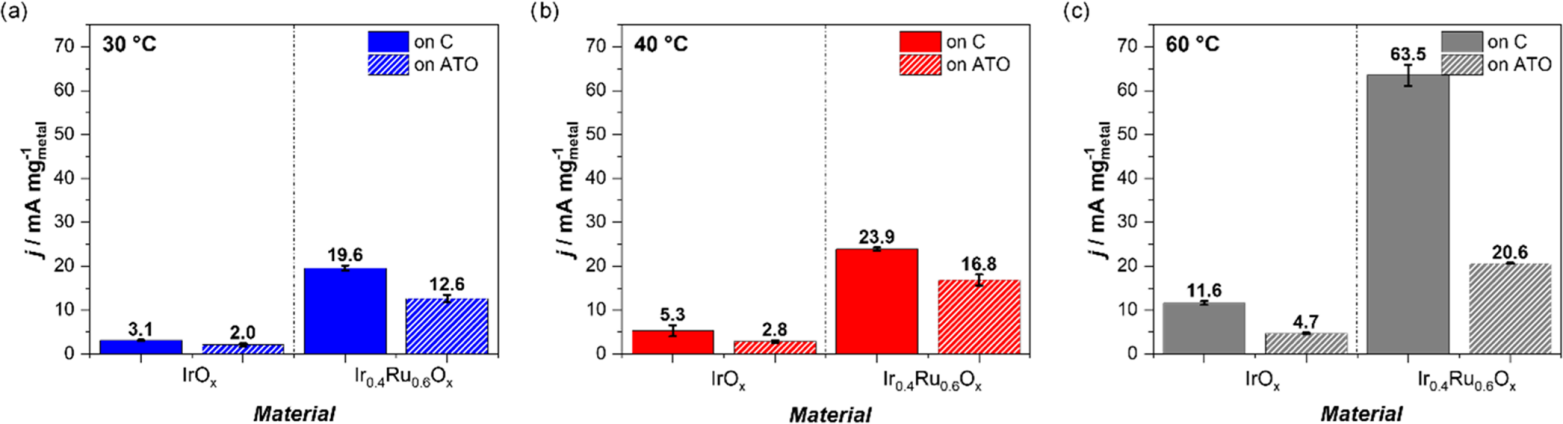
Comparison of the activities reached at a temperature-corrected OER overpotential η = 0.23 V for IrO*_x_* (left-hand side of the graphs) and Ir_0.4_Ru_0.6_O*_x_* (right-hand side of the graphs) deposited on C (solid bars) or ATO (dashed bars) at 30 (a), 40 (b), and 60 °C (c). Values were interpolated or extrapolated based on their Tafel slopes.

As already shown in our previous OER GDE study,^42^ and as expected from kinetics, increasing the temperature leads to an improved catalytic activity (lower OER overpotential). The catalytic activity of the four different catalysts is compared at an identical overpotential of η = 0.23 V in Figure 4a–c. This overpotential was chosen as it lies in the linear Tafel region of the individual catalysts at most temperatures. However, for IrO*_x_*/C at 30 °C and IrO*_x_*/ATO at 30 and 40 °C, the OER activities are obtained *via* extrapolation of the measured data. Note further that the temperature dependence of the reversible potential was corrected according to Parthasarathy et al.^62^ for the conversion of one mol of water (*n* = 2). Moreover, the performance of the catalysts is also compared in Figure S11 at a fixed current density of 25.68 mA mg_metal_^–1^, which is close to the benchmark value of 10 mA cm_geo_^–2^.

From the comparison, it can be seen at first glance that Ru-containing catalyst always exhibits a higher catalytic mass activity (total metal mass) as compared with pure Ir. This agrees with an early study of Kötz et al.,^63^ which suggested that combining Ru with Ir not only leads to better stability of Ru but also to an improved activity as compared with pure Ir. Note that EDX analysis of our Ir_0.4_Ru_0.6_ catalyst films indicates that during activation, part of the Ru is leached from the alloy nanoparticles (see Table S8). Despite this leaching of Ru, our data reveal that at 30 °C, the Ru-containing catalysts have a 6.3-fold higher OER activity than the pure Ir catalysts, independently of the support. The superior activity of Ir_0.4_Ru_0.6_ as compared with pure Ir is also confirmed at higher temperatures, where dependent on support and temperature, improvement factors between *ca.* 4.5- and 6-fold are observed.

As recently reported by Suermann et al.,^64^ Hartig-Weiss et al.,^15^ and Schröder et al.,^42^ the apparent OER activation energy (E_a_) can be approximated using the linearization of the Arrhenius equation (eq 2),
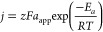
2where *j* is the current density, z is the number of electrons exchanged, *F* is the Faraday constant, *a*_app_ is the apparent preexponential factor that includes all of the entropic terms, *E*_*a*_ is the apparent activation energy, *R* is the gas constant, and *T* is the temperature. eq 2 can be used when similar Tafel slopes are obtained at different temperatures. We calculated the apparent OER activation energy *E_a_* for the four catalysts at η = 0.23 V. IrO*_x_*/C and Ir_0.4_Ru_0.6_O*_x_*/C exhibit an apparent *E_a_* equal to 37 and 34 kJ mol^–1^, respectively, which is in line with the work of Hartig-Weiss et al.^15^ On the other hand, ATO-supported catalysts exhibit much lower apparent activation energy, *i.e.*, 23 and 13 kJ mol^–1^ for IrO*_x_* and Ir_0.4_Ru_0.6_O*_x_*, respectively. It must be emphasized that these obtained values correspond to the apparent activation energy, and therefore are artificial. A recent study by Duan et al.^65^ on alkaline OER describes different factors that can lead to deviations in the activation energy. One of them is the change of active sites under operating conditions. However, our data indicate a different cause, which is support-related, for the apparent lower activation energy, *i.e.*, that ATO is not stable under operation conditions.

Comparing the support’s influence on the catalytic performance of the NPs in Figure 4, it can be seen that in all investigated cases, the NPs immobilized on C were more active than the ones immobilized on ATO. As discussed above, according to the characterization, the supported NPs show a similar size distribution, crystalline structure, and elemental ratio. The TEM micrographs indicate even better particle distribution on ATO than on the carbon support. Therefore, one would assume that the activity of the NPs would be identical regardless of the support, or that the performance of the carbon-supported NPs would be inferior due to carbon corrosion. However, the opposite is observed. Major contributions to the recorded current from the carbon support oxidation seem unlikely, although it cannot be excluded that carbon corrosion takes place. However, in contrast to the typical activity determination *via* potential scans, in the quasi-steady-state measurements, it would lead to time-dependent behavior (similar to what is seen during the activation) and to a nonlinear Tafel slope.^31^ Furthermore, as discussed above, the activation energy of the carbon-supported samples compares well with the literature data.^15^ Therefore, the most plausible cause for this observation is a higher conductivity/stability of the carbon support (9.85 S cm^–1^) as compared with the ATO (0.0009 S cm^–1^) (see Table S9) (note that it has been avoided to expose the catalyst to reducing conditions during the electrochemical measurements). In particular, our data suggest that going to elevated temperature diminishes the performance of the ATO-supported samples. In the literature, there is still an ongoing debate about whether or not ATO loses its conductivity under operating conditions.^66−73^ While some researchers did not observe any conductivity loss of their homemade mesoporous ATO after 15 h at 1 mA cm^–2^,^69^ others detected the loss of Sb in a commercially available ATO when sweeping the potential from open-circuit potential (OCP) to 2 V *vs* RHE.^70^ Determining the average Sn:Sb ratio by EDX in our pristine and postmortem samples of the catalysts supports this hypothesis (see Figures S12 and S13 and Table S10). Moreover, according to da Silva et al.,^71^ the doping of their homemade SnO_2_ does not significantly improve the activity of the catalyst. On the contrary, for hydrous IrO*_x_*, the dopants accelerate the dissolution of Ir and SnO_2_. The contradicting reports might be related to experimental limitations. Typically, OER studies are performed in conventional RDE measurements, where milder conditions are applied compared with MEAs or stack electrolyzers. Furthermore, only thin catalyst films are investigated that are deposited on conducting working electrode disks. The GDE setup used in this study mimics more realistic and practical conditions by using a highly acidic electrolyte, higher loading, and higher temperature.^74^ Furthermore, higher current densities can be applied without massive and detrimental oxygen bubble formation. In summary, our data suggest that the observed activity trend between carbon and ATO stems from the leaching of Sb in the ATO support, which is promoted at elevated temperatures.

## 4 Conclusions

In the present study, we evaluated four different catalysts for the OER under acidic conditions, *i.e.*, IrO*_x_*/C, Ir_0.4_Ru_0.6_O*_x_*/C, IrO*_x_*/ATO, and Ir_0.4_Ru_0.6_O*_x_*/ATO. The catalysts were synthesized in two steps using a straightforward route that allows independent optimization of single components such as the ratio between the metals, the nature of the support material, and the metal loading. The pristine (∼1.5 nm) NPs were immobilized on carbon as well as on ATO. Activation at 1.6 V for 5 min leads to oxide formation and corresponding particle growth but is not sufficient for the complete oxidation of the catalysts, which is only reached during the activity measurements. The performance of the different catalysts was investigated using a GDE setup in a galvanostatic operation mode. Ir_0.4_Ru_0.6_O*_x_*/C exhibits the highest activity at η = 0.23 V among the four presented catalysts. Excellent performance of 63.5 mA mg_metal_^–1^ was achieved at 60 °C, a temperature close to realistic conditions in PEMWE. The determined performance can be seen as the intrinsic OER activity of the Ir_0.4_Ru_0.6_O*_x_* NPs and thus is of interest for applications. Also, the determined apparent activation energy was within promising values of 34–37 kJ mol^–1^. The main challenge, however, remains a suitable catalyst support material. Our measurements clearly indicate that the employed commercial ATO is not a feasible support material, similar to carbon, which is not a viable option for industrial applications. In fact, ATO is inferior to carbon, despite high applied current densities and elevated temperatures. In particular, elevated temperatures lead to diminishing performance of the ATO-supported catalysts. This observation was suspected to be caused by a loss of conductivity due to Sb leaching. In consequence, further investigations need to be taken to design more suitable supports for OER catalysts. Moreover, these supports need to be tested under more realistic conditions to reveal their possible commercial applicability.

## Supporting Information Available

Chemicals and materials, loading determination by TGA, GDE setup sketch, TEM micrographs of nonsupported particles and their size distribution, XAS of pristine Ir/C and Ir/ATO, PDF of the pristine and activated Ir/C and IrRu/C, EXAFS of Ir and IrRu samples, particle size distribution by TEM and SAXS, PDF of activated Ir/ATO, potential transients and Tafel slopes of Ir/C and Ir/ATO, comparison histograms at 25.68 mA mg_metal_^–1^, summary of EDX data, as well as conductivity measurements (PDF)
