# Supplementary material for: Influence of Temperature on the Performance of Carbon- and ATO-supported Oxygen Evolution Reaction Catalysts in a Gas Diffusion Electrode Setup
Source: ACS Catal. 2023 May 22;13(11):7568–77. doi: 10.1021/acscatal.3c01193 (PMC10242686; doi:10.1021/acscatal.3c01193)
Supplement: Supplementary file 1 — cs3c01193_si_001.pdf [file cs3c01193_si_001.pdf]

Supporting information

Influence of temperature on the performance of carbon- and ATO-supported OER catalysts in a GDE setup

Aline Bornet[1], Rebecca Pittkowski[2], Tobias M. Nielsen[2], Etienne Berner[1], Annabelle Maletzko[3], Johanna Schröder[1], Jonathan Quinson[2,4], Julia Melke[3], Kirsten M. Ø. Jensen[2], Matthias Arenz[1,*]

[1] Department of Chemistry, Biochemistry and Pharmaceutical Sciences, University of Bern, Freiestrasse 3, 3012 Bern, Switzerland

[2] Department of Chemistry, University of Copenhagen, Universitetsparken 5, 2100 Copenhagen, Denmark

[3] Department for Applied Electrochemistry, Fraunhofer-Institute for Chemical Technology ICT, Joseph-von-Fraunhofer Strasse 7, 76327 Pfinztal, Germany

[4] Biochemical and Chemical Engineering Department, Aarhus University, Åbogade 40, 8200 Aarhus, Denmark

* Corresponding authors: matthias.arenz@unibe.ch

Chemicals and materials

Ultra-pure water (MilliQ-system, 2.7 ppb total organic carbon (TOC), 18.2 MΩ) was used to clean the GDE cell and to prepare the electrolyte and the catalyst ink.

For the catalyst synthesis, hydrated $IrCl_3$ (99.8% metals basis) and $RuCl_3$ (ReagentPlus) were purchased from Alfa Aesar and Sigma Aldrich, respectively, and stored in a glovebox. EtOH (EtOH absolute, VWR Chemicals) was used to dissolve the precursor salt, to prepare the alkaline (NaOH, Hänseler) solvent, and to disperse the support carbon Ketjen black (EC-300J, Fuel Cell Store) and ATO (NanoArc, 99.5%, Alfa Aesar). The catalyst inks were prepared using isopropanol (IPA, HPLC grade, VWR Chemicals), KOH (Hänseler) and a Nafion dispersion in H_2O (D1021, Fuel Cell Store). A horn sonicator (Q500 sonicator, QSONICA sonicators) was used to disperse the support and the catalyst. A rotary evaporator (RC 600) from knf was employed to evaporate the solvent.

The GDEs were prepared using a coated gas diffusion layer (GDL, Freudenberg H23C8, 0.230 mm thick, Fuel Cell Store) and a Nafion membrane (Nafion 117, 183 µm thick, Fuel Cell Store). H_2O_2 (30%, Hänseler) and H_2SO_4 (96%, Grogg Chemie) were used to activate the Nafion membrane (see below). The assembly of the GDE setup needed also a non-coated GDL (Freudenberg H23, 0.210 mm thick, Fuel Cell Store). The electrolyte was prepared using $HClO_4$ (ACS reagent, 70%) from Sigma Aldrich. O_2 (Alphagaz 1, 45) was purchased from Air Liquide. The electrochemical measurements were performed with a computer controlled Nordic electrochemistry potentiostat (ECi – 242).

For conductivity measurements, a Keithley 2400 multimeter and a laser distance sensor LAR-10-5V from Waycon Präzisionstechnik GmbH were used.

Cu grids (for unsupported catalyst: Quantifoil, 100 Classic Holey Carbon films; for supported catalysts: Quantifoil, 100 Lacey Carbon films) were used as sample holder for the TEM characterization. For SEM/EDX characterization, Cu tape (3M #1182 electrical tape) was used as a conductive adhesive tape. Graphite foil (0.13 mm thick, 99.8%, Alfa Aesar) was used as

sample holder for EDX characterization of the inks. Polyimide tubes (Wall thickness: 0.025 mm, outside diameter 1.05 mm, GoodFellow) were used as sample holders for total scattering experiments at DanMax beamline at MAXIV synchroton.

Nafion membrane activation

⌀ 2 cm Nafion membranes were punched out of a Nafion sheet. The ⌀ 2 cm membrane were treated at 80 °C for 30 min in 5 wt.% H_2O_2. Then, they were rinsed with excess of MilliQ water and heated in MilliQ water at 80 °C for 30 min. After rinsing them, they were treated with 8 wt.% H_2SO_4 at 80 °C for 30 min. Finally, the activated membranes were rinsed with excess of MilliQ water and stored in MilliQ water.

WE loading determination by TGA

Table S1. Summary of TGA data of Ir NPs immobilized on C-support measured in O_2-atmosphere between 25 and 1000 °C at 10 °C min^{-1}. The expected metal loading on the GDE is 1 mg_{Ir} cm^{-2}.

	m_i(Ir) / mg	Loading(Ir) / mg_{Ir} cm^{-2}	m(IrO_2) / mg	Loading(IrO_2) / mg_{IrO2} cm^{-2}
Expected values	0.071	1.000	0.082	1.166
Measured values	/	/	0.061	0.863

As the measurement was performed in an oxidative environment at elevated temperatures, a full oxidation of the Ir nanoparticles to IrO_2 is expected at the end of the experiment. The relative error to the expected loading is 26%. It must be noted that the initial mass of Ir is a nominal value (calculated from a total reduction of $IrCl_3$ during the synthesis).

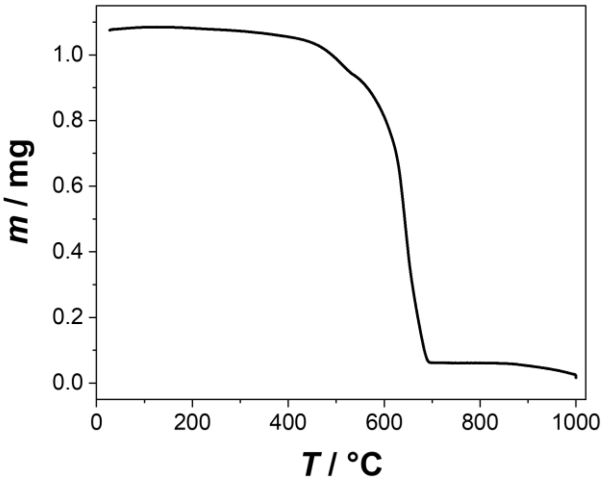

Figure S1. TGA curve of Ir NPs immobilized on C-support measured in O_2-atmosphere between 25 and 1000 °C at 10 °C min^{-1}.

GDE setup and its assembly

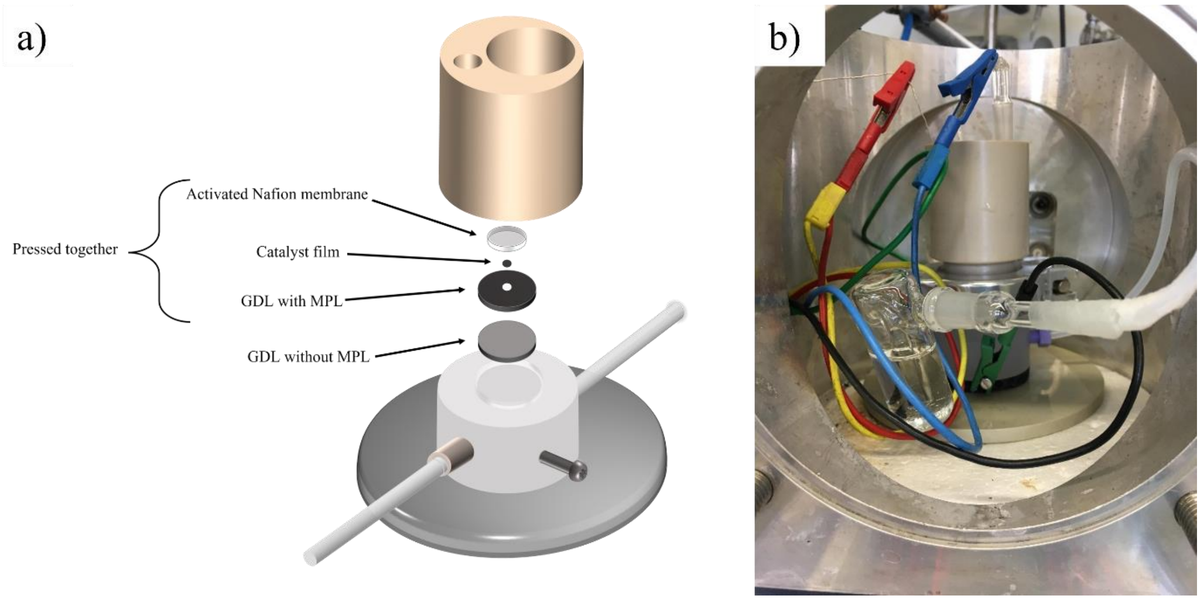

Figure S2. Sketch of the GDE setup assembly (a) (image adapted from Schröder *et al.*[1] (a) and picture of the connected GDE setup in a Faradaic cage (b).

Synthesis of different composition and TEM micrographs of the different compositions

The synthesis of the nominal composition of $Ir_{0.66}Ru_{0.33}$, $Ir_{0.33}Ru_{0.66}$ and Ru NPs follow the same approach than the one described in the experimental section **a**. Only the pipetted ratio between 20 mM $IrCl_3$ in EtOH and 20 mM $RuCl_3$ in EtOH differ and are the following:

- For $Ir_{0.66}Ru_{0.33}$ NPs: 6.67 mL of 20 mM $IrCl_3$ in EtOH and 3.33 mL 20 mM $RuCl_3$ in EtOH,
- For $Ir_{0.33}Ru_{0.66}$ NPs: 3.33 mL of 20 mM $IrCl_3$ in EtOH and 6.67 mL 20 mM $RuCl_3$ in EtOH, and
- For Ru NPs: 2 mL of 20 mM $RuCl_3$ in EtOH.

The mixtures were refluxed to 95 °C for 10 min to obtain $Ir_{0.66}Ru_{0.33}$ and $Ir_{0.33}Ru_{0.66}$, respectively 30 min to obtain pure Ru NPs.

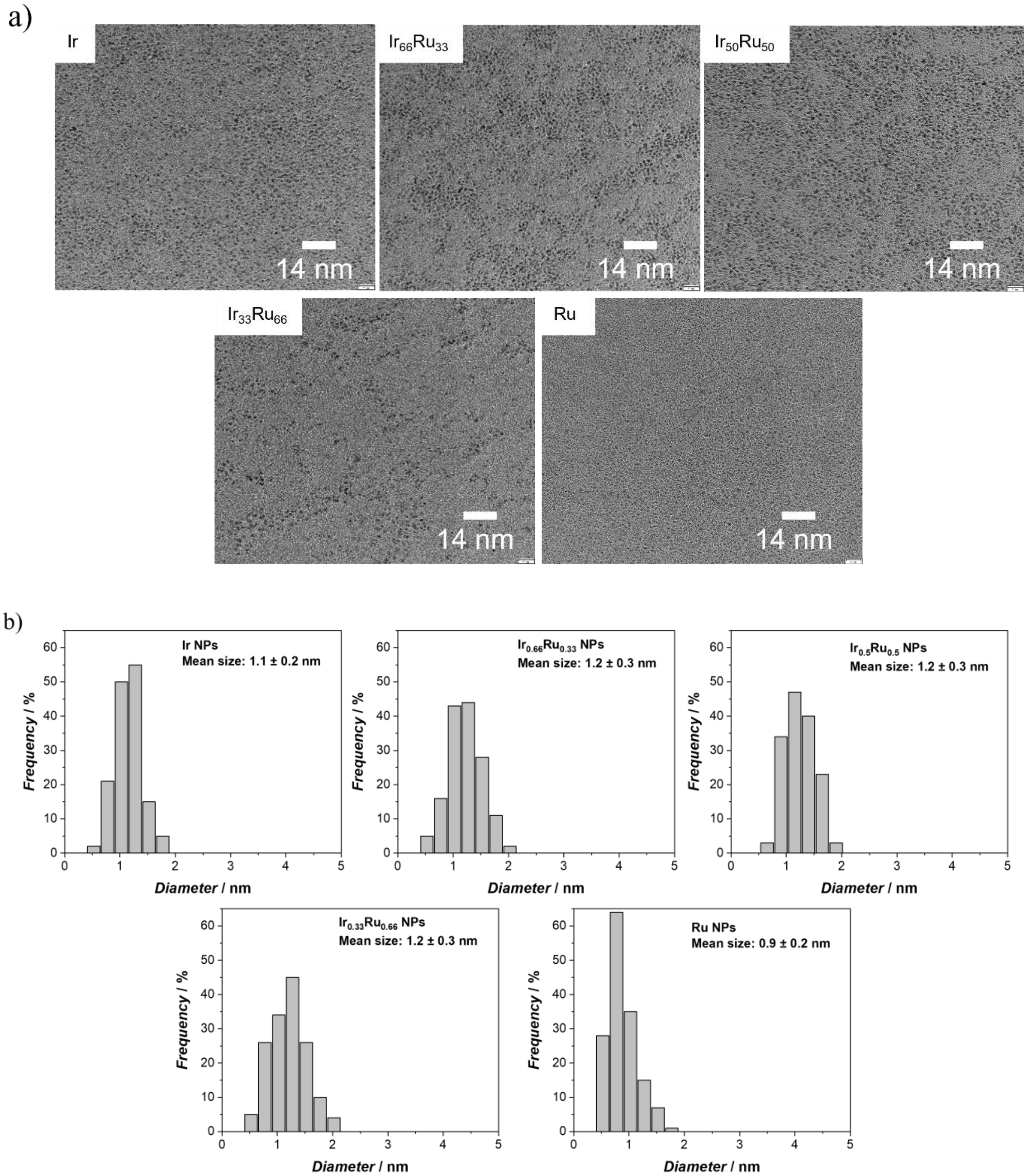

Figure S3. TEM micrographs of different nominal compositions of unsupported Ir_xRu_y NPs (a) and their respective size distribution (b).

XAS of pristine Ir/C and Ir /ATO – influence of the support on the structure of the NPs

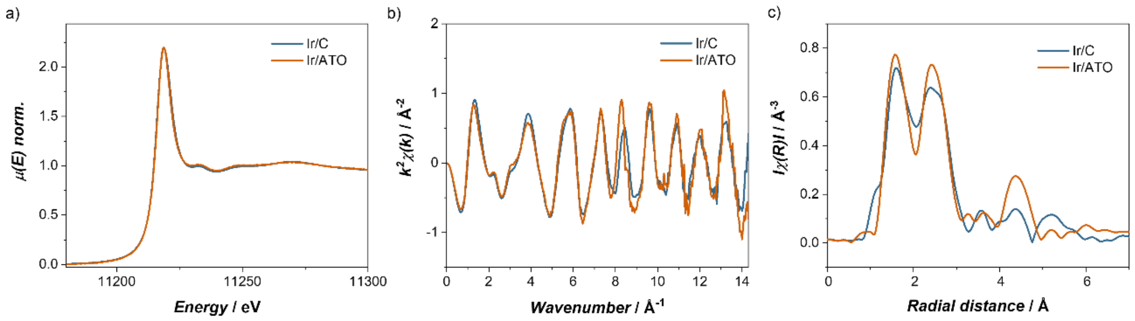

Figure S4. XANES spectra (a) and Fourier transform magnitudes of the k^2-weighted extended X-ray absorption fine structure (EXAFS) (b) and (c) data of pristine Ir/C and Ir/ATO measured at the Ir L_{III}-edge.

As both the Iridium XANES spectra and the EXAFS of the NPs supported on C and ATO agree, we find no influence of the support on the structure of the metal NPs. This is further supported in the modelling of the EXAFs data, see Table S2.

Table S2. Parameters obtained for EXAFS data fitting of pristine Ir/C and Ir/ATO on the Ir L_{III}-edge, that shows first nearest neighbour coordination shell (N), atomic bond length (R), Debye Waller factors (mean squared bond length disorder) (σ^2), absorption edge energy (E_0), and R_f-factor as a measure of fit quality.

**Ir edge of pristine samples**						
Sample	Bond	N	R / Å	σ^2 / Å^2	E_0 / eV	R_f
Ir/C	Ir-O_1	3.0 ± 0.4	1.99(2)	0.006(2)	11225 ± 1	0.008
	Ir-Ir_1	6.4 ± 0.6	2.70(2)	0.005(4)		
Ir/ATO	Ir-O_1	2.5 ± 0.4	1.99(2)	0.002(2)	11225 ± 3	0.019
	Ir-Ir_1	5.9 ± 2.0	2.71(1)	0.004(2)		

Aging process monitored by XAS

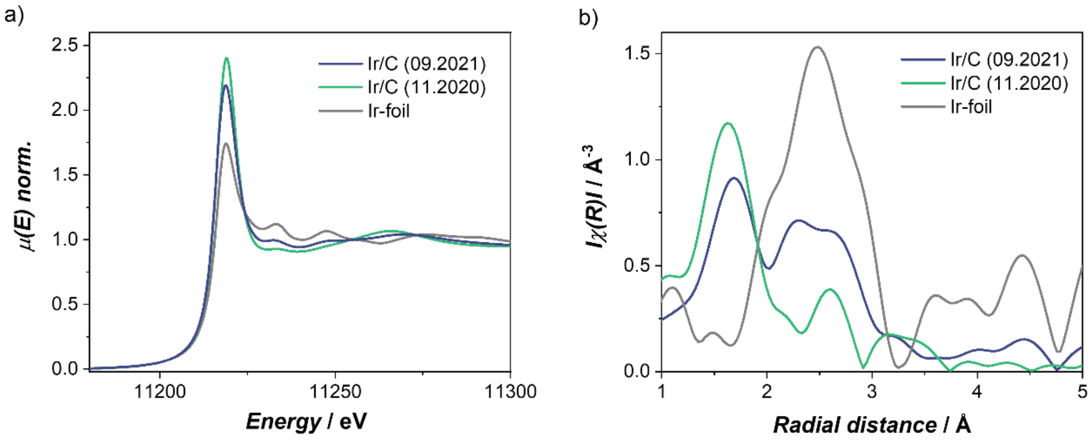

Figure S5. Effect of storage on the degree of oxidation of the catalyst (Ir/C) film stored in the air. XANES spectra (a) and Fourier transform magnitudes of the k^2-weighted extended X-ray absorption fine structure (EXAFS) data (b) of differently aged Ir/C and an Ir metal foil reference measured at the Ir L_{III}-edge. The Ir/C film corresponding to the green line was prepared 15 months prior and the one corresponding to the blue line only 5 months prior the measurements performed in February 2022.

PDF analysis of pristine and activated C-immobilized catalysts

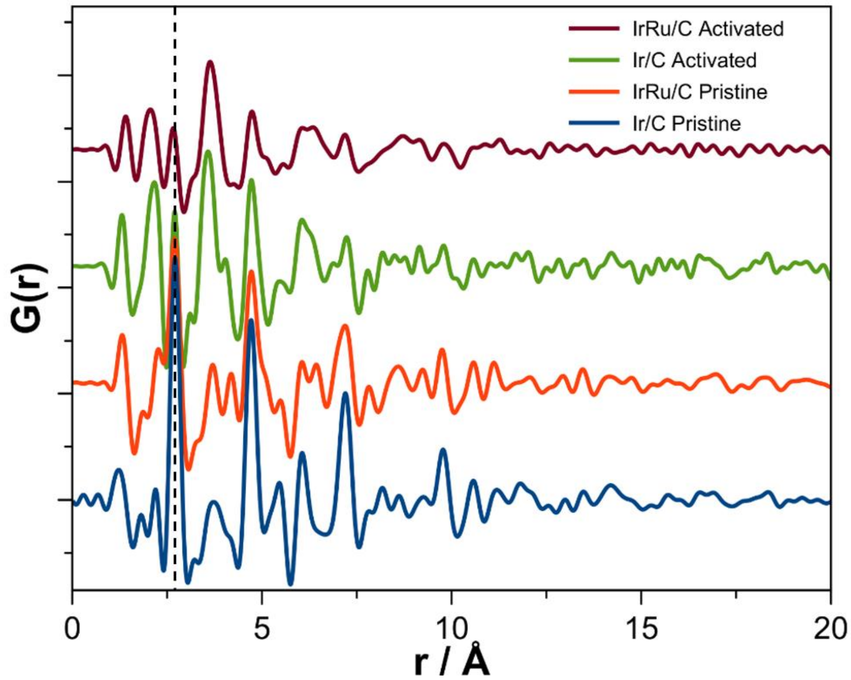

Figure S6. PDF of the total scattering experiments on C-immobilized Ir and $Ir_{0.4}Ru_{0.6}$, both pristine and activated. All data are background subtracted.

The peak at *ca.* 3.6 Å is more intense for the activated samples than for the pristine ones. This peak arises from a metal-oxygen distance in the structure, showing the presence of the oxide phase in the material. At *ca.* 2.7 Å (dotted line), the peak (metal-metal distance) of the activated samples losses its intensity, but do not vanish, thus, confirming the coexistence of both metallic and oxide species in the activated samples.

Table S3. Summarized particle sizes, unit cell parameter and atomic displacement parameter (ADP) for all pristine and activated Ir and $Ir_{0.4}Ru_{0.6}$ catalyst immobilized on carbon-support. For the pristine $Ir_{0.4}Ru_{0.6}$ sample, both a fcc Ir phase and a hcp Ru phase were included to get the best fit. The quality of the fits was determined by the R_w values and are R_w= 0.33 for Ir/C, R_w= 0.69 for IrO_2/C, R_w= 0.62 for the $Ir_{0.4}Ru_{0.6}$/C and R_w= 0.57 for $Ir_{0.4}Ru_{0.6}O_x$/C.

**Ir/C pristine – Ir fit**	
Particle size	15 Å
Unit cell parameter a, b, c	3.85 Å
ADP	0.00783 Å^2
**IrO_x /C activated – Ir fit**	
Particle size	14 Å
Unit cell parameter a, b, c	3.86 Å
ADP	0.002865 Å^2
**IrO_x/C activated – IrO_2 fit**	
Particle size	7 Å
Unit cell parameter a, b	4.59 Å
Unit cell parameter c	3.18 Å
ADP for Ir	0.009216 Å^2
ADP for O	0006872 Å^2
**$Ir_{0.4}Ru_{0.6}$/C pristine – Ir fit**	
Particle size	14 Å
Unit cell parameter a, b, c	3.84 Å
ADP	0.01276 Å^2
**$Ir_{0.4}Ru_{0.6}$/C pristine – Ru fit**	
Particle size	36 Å
Unit cell parameter a, b	2.88 Å
Unit cell parameter c	3.99 Å
ADP	0.03846 Å^2
**$Ir_{0.4}Ru_{0.6}O_x$/C activated – Ir fit**	
Particle size	13 Å
Unit cell parameter a, b, c	3.85 Å
ADP	0.002283 Å^2
**$Ir_{0.4}Ru_{0.6}O_x$/C activated – IrO_2 fit**	
Particle size	7 Å
Unit cell parameter a, b	4.63 Å
Unit cell parameter c	3.44Å
ADP for Ir	0.006167 Å^2
ADP for O	0.004166 Å^2

EXAFS of pristine $Ir_{0.4}Ru_{0.6}$/C and $Ir_{0.4}Ru_{0.6}$/ATO – alloy determination

Table S4. Parameters obtained for EXAFS data fitting of pristine $Ir_{0.4}Ru_{0.6}$/C and $Ir_{0.4}Ru_{0.6}$/ATO on the Ir L_{III}-edge and the Ru K-edge, that shows first nearest neighbour coordination shell (N), atomic bond length (R), Debye Waller factors (mean squared bond length disorder) (σ^2), absorption edge energy (E_0), and R_f-factor as a measure of fit quality.

**$Ir_{0.4}Ru_{0.6}$/C pristine**						
Edge	Bond	N	R / Å	σ^2 / Å^2	E_0 / eV	R_f
Ir	Ir-O_1	2.7 ± 0.8	2.00(2)	0.007(4)	11226 ± 2	0.019
	Ir-Ru_1	3.2 ± 1.3	2.69(2)	0.004(3)		
	Ir-Ir_1	2.8 ± 1.2	2.69(2)	0.003(2)		
Ru	Ru-O_1	2.6 ± 0.7	2.00(2)	0.002(1)	22119 ± 3	0.010
	Ru-Ru_1	2.2 ± 0.3	2.68(2)	0.002(3)		
	Ru-Ir_1	3.0 ± 0.5	2.69(2)	0.002(3)		
**$Ir_{0.4}Ru_{0.6}$/ATO pristine**						
Ir	Ir-O_1	3.1 ± 2.4	2.00(2)	0.002(3)	11225 ± 4	0.055
	Ir-Ru_1	2.4 ± 1.3	2.67(4)	0.007(5)		
	Ir-Ir_1	2.8 ± 0.5	2.67(4)	0.007(6)		
Ru	Ru-O_1	4.1 ± 1.1	2.00(1)	0.007(4)	22119 ± 4	0.002
	Ru-Ru_1	1.3 ± 0.6	2.66(1)	0.005(7)		
	Ru-Ir_1	2.9 ± 2.5	2.68(1)	0.005(7)		

Particle size determination by TEM and SAXS of the supported catalysts

Table S5. Summarized diameter obtained by averaging particles sized for 150 NPs on TEM micrographs and by analysing SAXS data of the four supported catalysts.

Sample	TEM	SAXS	
	⌀, pristine sample / nm	⌀, pristine sample / nm	⌀, activated sample / nm
Ir/C	1.3 ± 0.4	1.8 ± 0.7	2.6 ± 1.3
$Ir_{0.4}Ru_{0.6}/C$	1.5 ± 0.4	2.8 ± 1.1	2.8 ± 1.3
Ir/ATO	1.3 ± 0.3	1.8 ± 0.8	2.8 ± 0.1
$Ir_{0.4}Ru_{0.6}/ATO$	1.3 ± 0.3	1.7 ± 0.7	2.7 ± 0.2

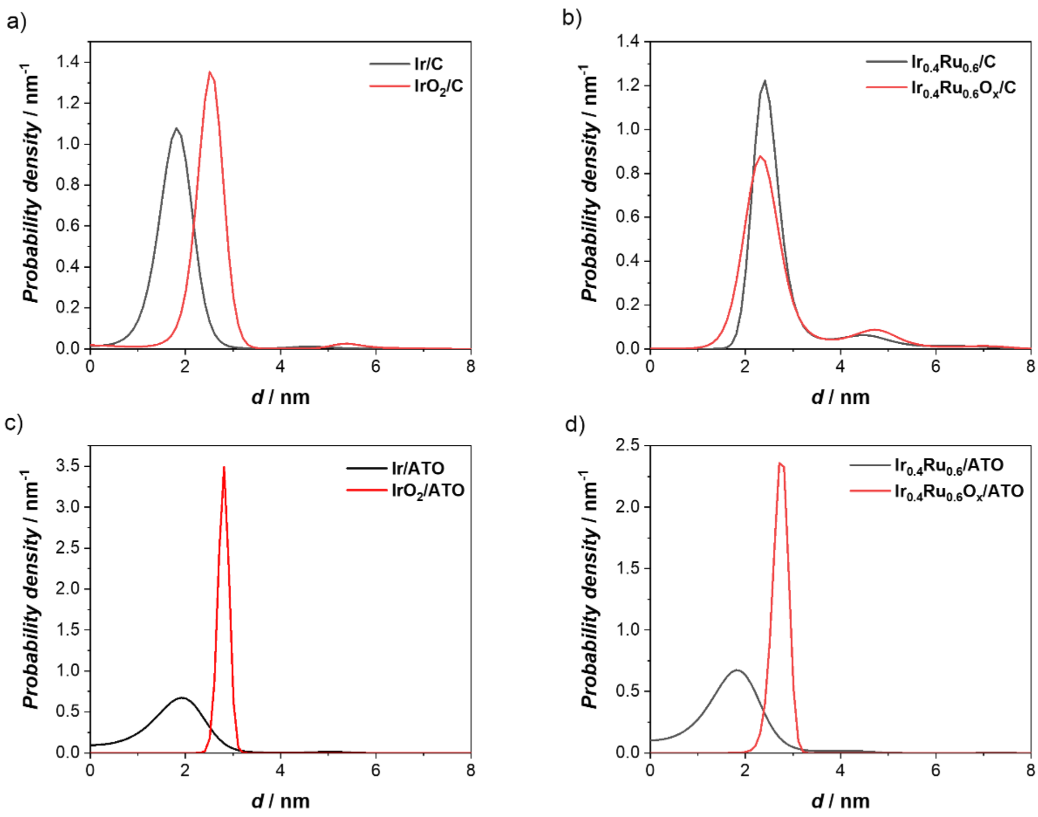

Figure S7. Particle size distribution monitored by SAXS for Ir/C (a), $Ir_{0.4}Ru_{0.6}/C$ (b), Ir/ATO (c), and $Ir_{0.4}Ru_{0.6}/ATO$ (d). The pristine (black) and the activated (red) samples are compared.

All samples beside $Ir_{0.4}Ru_{0.6}/C$ face a particle growth after the activation step. The reason behind the non-growth of $Ir_{0.4}Ru_{0.6}/C$ is explained by an aging process. The pristine film was prepared about a year prior the actual SAXS measurement, thus the NPs had time to oxidize in air. Therefore, both pristine and activated samples demonstrate a similar particle size.

Moreover, based on the density of Ir and IrO_2 (Ir: 22.6 g cm^{-3}, IrO_2: 2.00 g cm^{-3}) [2] and assuming fully reduced pristine species, a slightly higher than two-fold growth is expected if the entire NPs would be fully oxidized (from 1.8 to 4.0 nm). However, according to the obtained SAXS results, the particles grew only 1 nm (Table S5). This was explained by an incomplete oxidation of the NPs.

PDF analysis of activated Ir/ATO

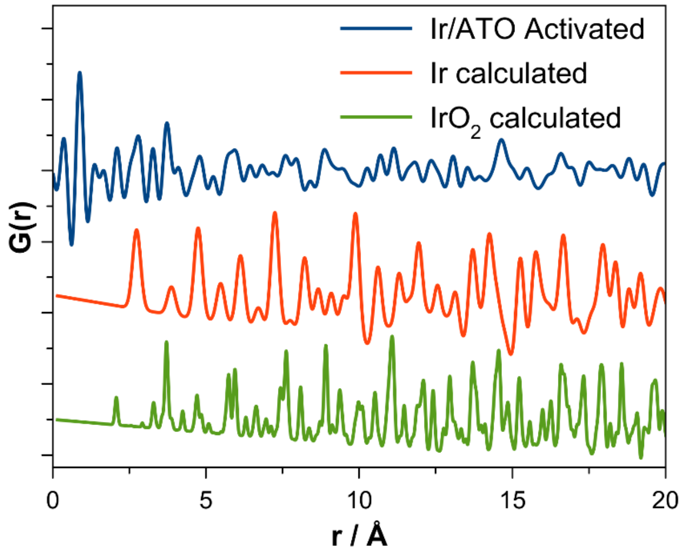

Figure S8. PDF of activated Ir/ATO. The peaks arising from either Ir or IrO_2 are difficult to distinguish due to the presence of the oxide support.

EXAFS of activated Ir/C and $Ir_{0.4}Ru_{0.6}/C$ – metal-metal coordination after activation

Table S6. Parameters obtained for EXAFS data fitting of activated Ir/C and $Ir_{0.4}Ru_{0.6}/C$ on the Ir L_{III}-edge and the Ru K-edge, that shows first nearest neighbour coordination shell (N), atomic bond length (R), Debye Waller factors (mean squared bond length disorder) (σ^2), absorption edge energy (E_0), and R_f-factor as a measure of fit quality.

**Ir/C activated**						
Edge	Bond	N	R / Å	σ^2 / Å^2	E_0 / eV	R_f
Ir	Ir-O_1	5.7 ± 0.7	2.02(1)	0.007(2)	11227 ± 2	0.016
	Ir-Ir_1	1.1 ± 0.9	2.70(1)	0.007(6)		
**$Ir_{0.4}Ru_{0.6}/C$ activated**						
Ir	Ir-O_1	5.7 ± 0.7	2.00(1)	0.006(2)	11227 ± 2	0.016
	Ir-Ru_1	0.3 ± 0.3	2.67(6)	0.004(6)		
	Ir-Ir_1	1.1 ± 0.9	2.68(3)	0.004(6)		
Ru	Ru-O_1	3.6 ± 0.5	1.92(1)	0.003(2)	22115 ± 3	0.045
	Ru-Ru_1	1.1 ± 0.4	2.66(1)	0.002(4)		
	Ru-Ir_1	2.9 ± 0.6	2.68(4)	0.002(4)		

i*R*-corrected potentials transients of IrO_x/C, IrO_x/ATO

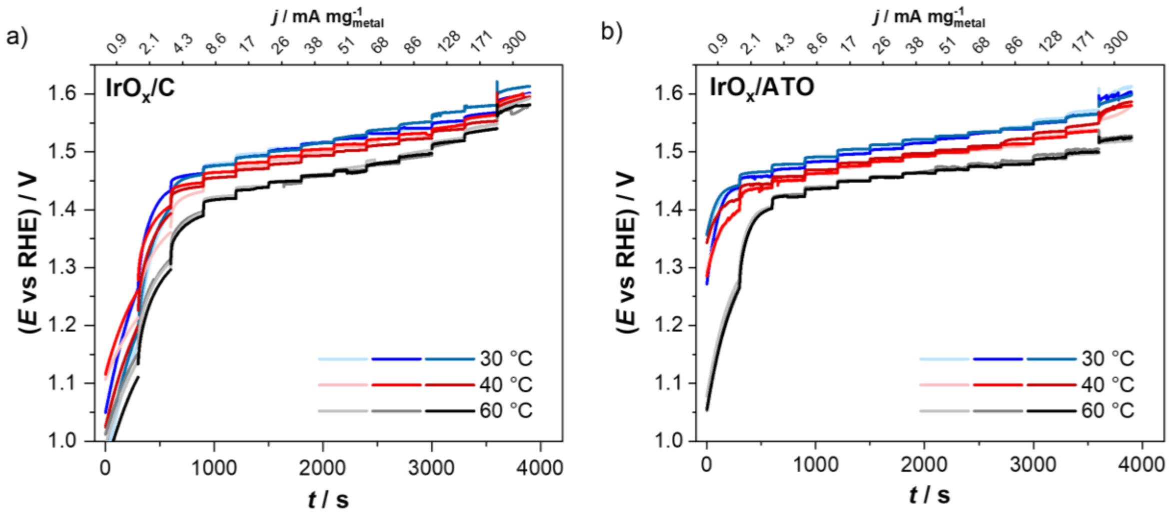

Figure S9. Electrocatalytic OER i*R*-corrected potential transients of IrO_x/C (a) and IrO_x/ATO (b) at 30 (blue), 40 (red) and 60 °C (black). All measurements were performed in the GDE setup in an O_2-atmosphere using 4 M $HClO_4$ as an electrolyte. Nominal catalyst loading: 654 μg_{metal} cm^{-2}.

Tafel plots and slopes of IrO_x/C and IrO_x/ATO

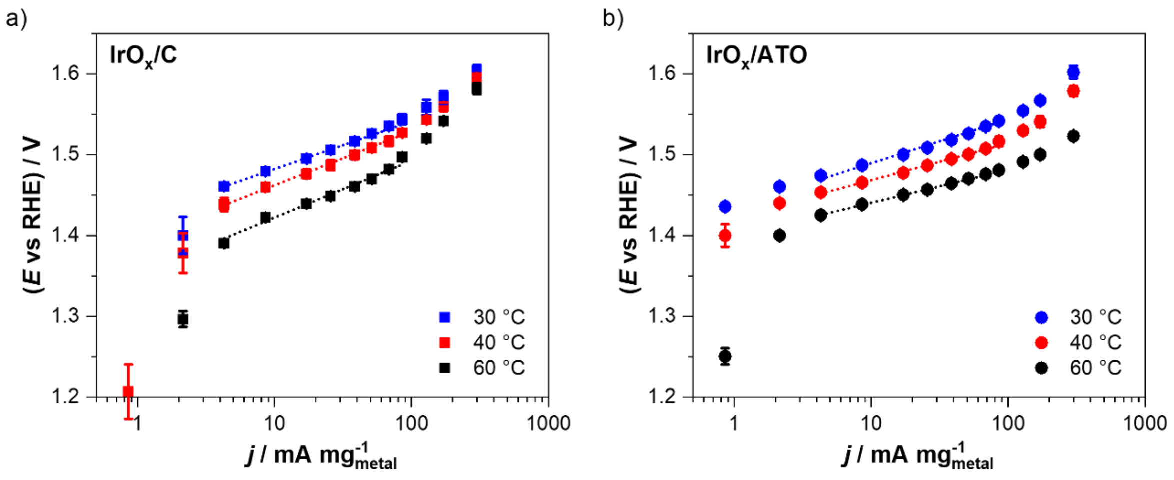

Figure S10. Tafel plots of IrO_x/C (a) and IrO_x/ATO (b) at 30 (blue), 40 (red) and 60 °C (black). The error bars show the standard deviation of the three independent measurements. All measurements were performed in the GDE setup in an O_2-atmosphere using 4 M $HClO_4$ as an electrolyte. Nominal catalyst loading: 654 μg_{metal} cm^{-2}.

Table S7. Tafel slopes of IrO_x/C, IrO_x/ATO, $Ir_{0.4}Ru_{0.6}O_x$/C and $Ir_{0.4}Ru_{0.6}O_x$/ATO at 30, 40 and 60 °C. The same mass current density range (ca. 4 and 85 mA mg_{metal}^{-1}) was chosen to determine the Tafel slopes.

Sample	Tafel slopes (mV dec^{-1}) at		
	30 °C	40 °C	60 °C
IrO_x/C	59	69	70
$Ir_{0.4}Ru_{0.6}O_x$/C	53	57	55
IrO_x/ATO	54	47	41
$Ir_{0.4}Ru_{0.6}O_x$/ATO	62	60	51

Comparison of OER activities measured at 30, 40, and 60 °C

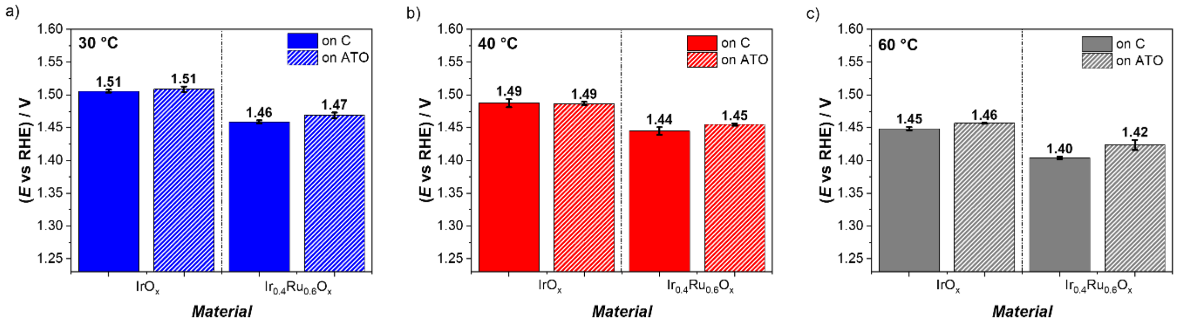

Figure S11. Comparison of the electrode potential reached at fixed a current density of 25.68 mA mg_{metal}^{-1} (which corresponds to ca. 7.5 mA cm^{-2}) for IrO_x (left hand side of the graphs) and $Ir_{0.4}Ru_{0.6}O_x$ (right hand side of the graphs) deposited on C (solid bars) or ATO (dashed bars) at 30 (a), 40 (b) and 60 °C (c).

EDX data – Ru leaching

Table S8. Summarized ratios (at.%) between Ir and Ru calculated by top-view EDX for $Ir_{0.4}Ru_{0.6}/C$ and $Ir_{0.4}Ru_{0.6}/ATO$ catalysts. The catalyst is in a form of an ink or a film.

Sample		Ratio / at.%	
		**Ir**	**Ru**
$Ir_{0.4}Ru_{0.6}/C$ pristine - ink		41	59
$Ir_{0.4}Ru_{0.6}/C$ pristine - film		41	59
$Ir_{0.4}Ru_{0.6}O_x/C$ activated at 60 °C - film	centre	41	59
	edges	64	36
$Ir_{0.4}Ru_{0.6}/ATO$ pristine - ink		41	59
$Ir_{0.4}Ru_{0.6}/ATO$ pristine - film		40	60
$Ir_{0.4}Ru_{0.6}O_x/ATO$ activated at 60 °C - film	centre	38	62
	edges	70	30

Activated Ru-containing samples at 60 °C were analysed to quantify any elemental leaching. It was found that Ru was partially and not homogenously leached out of the sample. The edges of the 3 mm sample showed a decrease of the Ru content, while the centre displayed an identical ratio as the pristine one.

Conductivity measurements

Table S9. Summarized ATO- and C-support individual conductivity measurements and their sample thickness. All measurements were performed with a mass of 100 mg and a pressure of 11.29 MPa.

Sample	Conductivity / S cm^{-1}	Sample thickness / mm
ATO support	9.67008 * 10^{-4}	1.23046
	7.96755 * 10^{-4}	1.16972
	9.11954 * 10^{-4}	1.21542
C support	9.72869	6.06978
	9.98393	5.89936

EDX data – Sb leaching

The suspected Sb leaching from the ATO support was determined *post-mortem* by EDX. It must be noted that the pristine and the *post-mortem* samples are different samples. In Fig. S11 and S12, the *post-mortem* samples measured at 30 °C and 60 °C for IrO_x/ATO and $Ir_{0.4}Ru_{0.6}O_x$/ATO are represented.

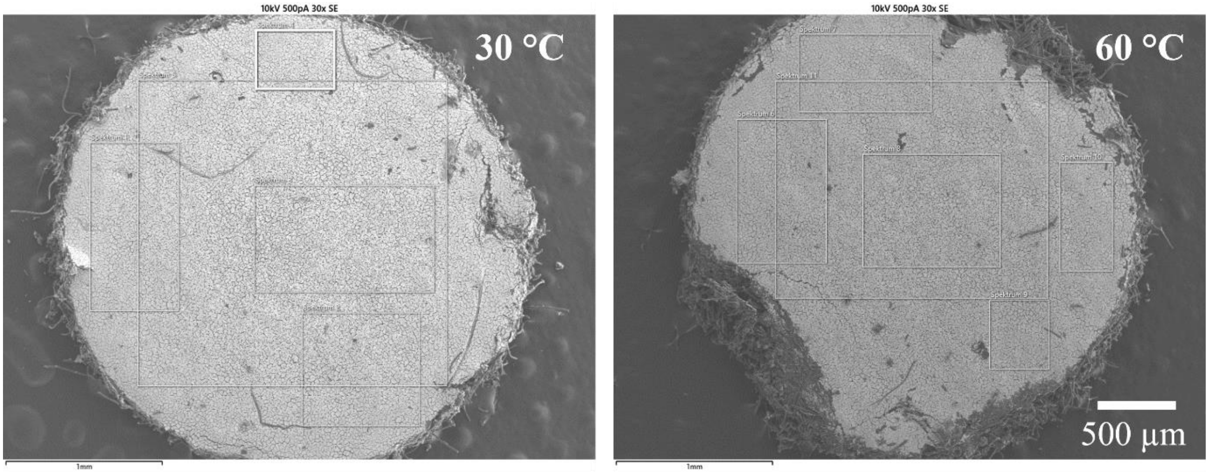

Figure S12. SEM images of the *post-mortem* IrO_x/ATO ⌀ 3 mm sample disks measured at 30 and 60 °C. The squares on the images correspond to the different areas analysed by EDX.

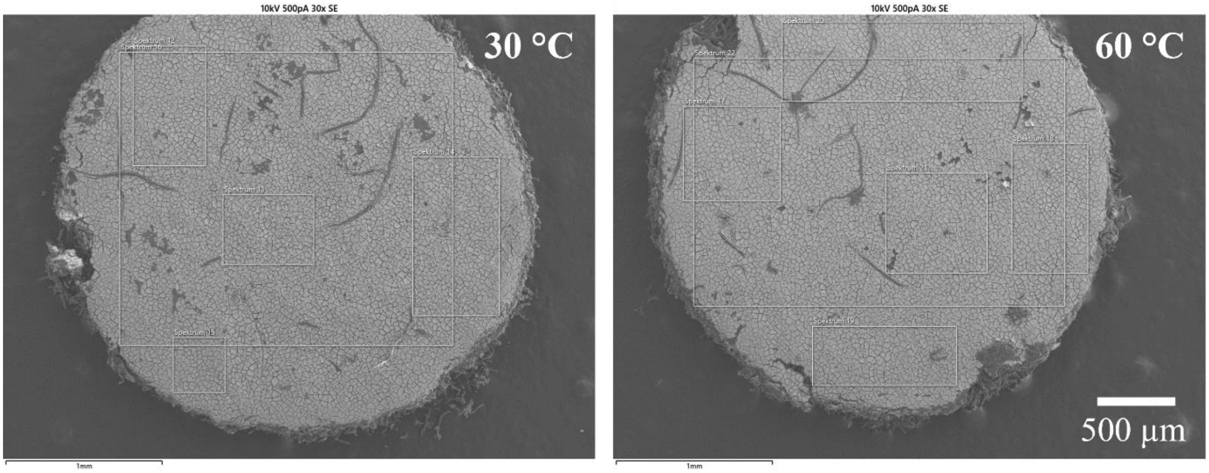

Figure S13. SEM images of the *post-mortem* $Ir_{0.4}Ru_{0.6}O_x$/ATO ⌀ 3 mm sample disks measured at 30 and 60 °C. The squares on the images correspond to the different areas analysed by EDX.

Table S10. Comparison of the ratios (wt.%) between Sn and Sb for pristine and *post-mortem* (30 and 60 °C) IrO_x/ATO and $Ir_{0.4}Ru_{0.6}O_x$/ATO catalyst films. The measurements were performed in a top-view configuration.

Sample	Area	Sn:Sb ratio (wt.%)	Average Sn:Sb ratio (wt.%)
Pristine Ir/ATO	/	/	87 : 13
Post-mortem IrO_x/ATO at 30 °C	1	90.5 : 9.5	89 : 11
	2	87.8 : 12.2	
	3	89.3 : 10.7	
	4	89.7 : 10.3	
	5	88.9 : 11.1	
Post-mortem IrOx/ATO at 60 °C	6	92.0 : 8.0	91 : 9
	7	90.2 : 9.8	
	8	90.3 : 9.7	
	9	90.4 : 9.6	
	10	91.0 : 9.0	
	11	89.6 : 10.4	
Pristine $Ir_{0.4}Ru_{0.6}$/ATO	/	/	85 : 15
Post-mortem $Ir_{0.4}Ru_{0.6}O_x$/ATO at 30 °C	12	87.4 : 12.6	87 : 13
	13	87.7 : 12.3	
	14	87.9 : 12.1	
	15	86.4 : 13.6	
	16	87.0 : 13.0	
Post-mortem $Ir_{0.4}Ru_{0.6}O_x$/ATO at 60 °C	17	87.7 : 12.3	88 : 12
	18	87.6 : 12.4	
	19	88.5 : 11.5	
	20	88.0 : 12.0	
	21	87.1 : 12.9	
	22	88.1 : 11.9	

References

(1) Schröder, J.; Mints, V. A.; Bornet, A.; Berner, E.; Fathi Tovini, M.; Quinson, J.; Wiberg, G. K. H.; Bizzotto, F.; El-Sayed, H. A.; Arenz, M. The Gas Diffusion Electrode Setup as Straightforward Testing Device for Proton Exchange Membrane Water Electrolyzer Catalysts. *JACS Au* **2021**, *1* (3), 247–251. https://doi.org/10.1021/JACSAU.1C00015.

(2) Hartig-Weiss, A.; Miller, M.; Beyer, H.; Schmitt, A.; Siebel, A.; Freiberg, A. T. S.; Gasteiger, H. A.; El-Sayed, H. A. Iridium Oxide Catalyst Supported on Antimony-Doped Tin Oxide for High Oxygen Evolution Reaction Activity in Acidic Media. *ACS Appl Nano Mater* **2020**. https://doi.org/10.1021/ACSANM.9B02230.
